# Maladie de Hoffa

**DOI:** 10.11604/pamj.2019.34.20.19441

**Published:** 2019-09-10

**Authors:** Taoufik Africha, Mohamed Ouahidi

**Affiliations:** 1 Service de Radiologie, Hopital Militaire My Ismail, Meknes, Maroc; 2 Service de Traumatologie, Hopital Militaire My Ismail, Meknes, Maroc

**Keywords:** Maladie de Hoffa, genou, IRM, Hoffa's disease, knee, MRI

## Abstract

We report the case of a 40-year female patient, with no previous pathological history, presenting with right unilateral mechanical gonalgias with progressive onset, which increased climbing and going down stairs associated with hydarthrosis on clinical examination. After normal standard X ray, MRI showed a grease of hypertrophied Hoffa's grease with heterogeneous hyposignal on T1 (A), hypersignal on T2 with grease saturation (B), heterogeneously enhancing after gadolinium injection. The diagnosis of Hoffa's disease was made and the patient, after failure of a medical treatment, underwent arthroscopy for ablation of the Hoffa's grease with good evolution. Hoffa's disease, a cause poorly known of pain of the anterior compartment of the knee, is an acute or chronic inflammation of the infrapatellar adipose tissue. The physiopathologic theory speaks of repeated micro traumas resulting in inflammatory, hemorrhagic and fibrous changes of the adipose body of Hoffa; at the final stage of the disease osteochondroma occurs. MRI is the test of choice in the early forms. It shows poorly limited edematous infiltrate with hypersignal on T2 and hyposignal on T1, in a hypertrophic infrapatellar fat body. Treatment is based on general or local non-steroidal anti-inflammatory drugs, icing, and physiotherapy, associated with immobilization. In the case of failure of conservative treatment or of osteochondroma, arthroscopic resection is the treatment of choice.

## Image en médecine

Patiente de 40 ans, sans antécédents, consulte pour gonalgies droites mécaniques unilatérales d'installation progressive, majorées à la descente et la montée des escaliers avec hydarthrose à l'examen clinique. Après une radiographie standard normale, l'IRM a montré une graisse de Hoffa hypertrophiée en hyposignal hétérogène T1 (A), en hypersignal T2 avec saturation de graisse (B), se rehaussant de façon hétérogène après injection de Gadolinium. Le diagnostic de maladie de Hoffa a été posé et la patiente, après échec d'un traitement médical, a été opérée sous arthroscopie pour ablation de la graisse de Hoffa avec bonne évolution. La maladie de Hoffa, cause mal connue de douleur du compartiment antérieur du genou, est une inflammation aiguë ou chronique du tissu adipeux infra-patellaire. La théorie physiopathologique parle de micro traumatismes répétés engendrant des remaniements inflammatoires, hémorragiques et fibreux du corps adipeux de Hoffa, au stade ultime de la pathologie apparaît un ostéochondrome. L'IRM est l'examen de choix dans les formes précoces, elle montre un infiltrat œdémateux mal limité, en hypersignal T2 et hypo-signal T1, dans un corps adipeux infrapatellaire hypertrophique.

**Figure 1:**
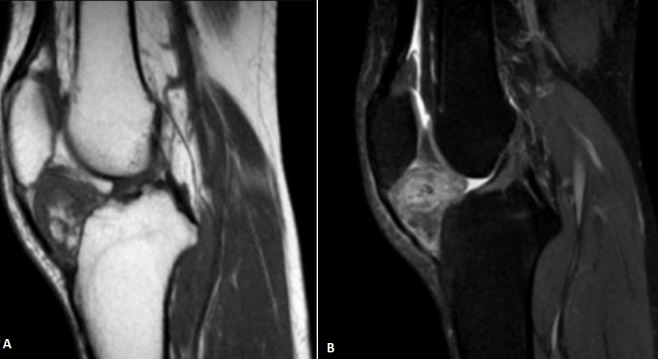
IRM du genou montrant une graisse de Hoffa hypertrophié en hyposignal hétérogène T1 (A), en hypersignal T2 avec saturation de graisse (B), se rehaussant de façon hétérogène après injection de Gadolinium

